# Evolutionary history and spatiotemporal dynamics of the HIV-1 subtype B epidemic in Guatemala

**DOI:** 10.1371/journal.pone.0203916

**Published:** 2018-09-13

**Authors:** Yaxelis Mendoza, Claudia García-Morales, Gonzalo Bello, Daniela Garrido-Rodríguez, Daniela Tapia-Trejo, Juan Miguel Pascale, Amalia Carolina Girón-Callejas, Ricardo Mendizábal-Burastero, Ingrid Yessenia Escobar-Urias, Blanca Leticia García-González, Jessenia Sabrina Navas-Castillo, María Cristina Quintana-Galindo, Rodolfo Pinzón-Meza, Carlos Rodolfo Mejía-Villatoro, Santiago Avila-Ríos, Gustavo Reyes-Terán

**Affiliations:** 1 Gorgas Memorial Institute for Health Studies, Panama City, Panama; 2 Department of Genetics and Molecular Biology, University of Panama, Panama City, Panama; 3 Centre for Research in Infectious Diseases, National Institute of Respiratory Diseases, Mexico City, Mexico; 4 Laboratório de AIDS e Imunologia Molecular, Instituto Oswaldo Cruz, FIOCRUZ, Rio de Janeiro, Brazil; 5 Universidad del Valle de Guatemala, Guatemala City, Guatemala; 6 Infectious Diseases Clinic, Roosevelt Hospital, Guatemala City, Guatemala; Public Health Agency of Canada, CANADA

## Abstract

Different explanations exist on how HIV-1 subtype B spread in Central America, but the role of Guatemala, the Central American country with the highest number of people living with the virus, in this scenario is unknown. We investigated the evolutionary history and spatiotemporal dynamics of HIV-1 subtype B in Guatemala. A total of 1,047 HIV-1 subtype B *pol* sequences, from newly diagnosed ART-naïve, HIV-infected Guatemalan subjects enrolled between 2011 and 2013 were combined with published subtype B sequences from other Central American countries (n = 2,101) and with reference sequences representative of the B_PANDEMIC_ and B_CAR_ lineages from the United States (n = 465), France (n = 344) and the Caribbean (n = 238). Estimates of evolutionary, demographic, and phylogeographic parameters were obtained from sequence data using maximum likelihood and Bayesian coalescent-based methods. The majority of Guatemalan sequences (98.9%) belonged to the B_PANDEMIC_ clade, and 75.2% of these sequences branched within 10 monophyletic clades: four also included sequences from other Central American countries (B_CAM-I_ to B_CAM-IV_) and six were mostly (>99%) composed by Guatemalan sequences (B_GU_ clades). Most clades mainly comprised sequences from heterosexual individuals. Bayesian coalescent-based analyses suggested that B_GU_ clades originated during the 1990s and 2000s, whereas B_CAM_ clades originated between the late 1970s and mid 1980s. The major hub of dissemination of all B_GU_, and of B_CAM-II,_ and B_CAM-IV_ clades was traced to the Department of Guatemala, while the root location of B_CAM-I_ and B_CAM-III_ was traced to Honduras. Most Guatemalan clades experienced initial phases of exponential growth (0.23 and 3.6 year^-1^), followed by recent growth declines. Our observations suggest that the Guatemalan HIV-1 subtype B epidemic is driven by dissemination of multiple B_PANDEMIC_ founder viral strains, some restricted to Guatemala and others widely disseminated in the Central American region, with Guatemala City identified as a major hub of viral dissemination. Our results also suggest the existence of different sub-epidemics within Guatemala for which different targeted prevention efforts might be needed.

## Introduction

It is commonly accepted that the HIV-1 subtype B epidemic likely moved out of Africa via a single introduction to Haiti in the mid 1960s [1]. This event was followed by a wide spread of phylogenetically distinct Caribbean-specific lineages (B_CAR_) throughout the Caribbean islands [1–3], with non-pandemic B_CAR_ lineages accounting for more than 40% of infections in the region [3]. A pandemic B lineage (B_PANDEMIC_), encompassing most subtype B infections around the world, subsequently emerged in the late 1960s [1], spreading from the Caribbean to North America and later expanding to other parts of the world [1, 4, 5], including possible re-introductions to the Caribbean [6].

The precise routes of introduction of HIV-1 subtype B to Central America remain to be clarified. One study suggests that a single early introduction of a B_PANDEMIC_ strain possibly to Honduras during the 1960s originated a Central American lineage (B_CAM_) that accounts for approximately 60% of current subtype B cases in the region [7]. Other sub-epidemics originating mostly during the 1970s and early 1980s were generated by the spread of additional founder viruses that tended to cluster according to the country of origin [6, 7]. A more detailed study in Panama, however, showed that the subtype B epidemic in this country is mostly driven by the expansion of multiple founder B_PANDEMIC_ and B_CAR_ strains, that have remained mostly restricted to this country [8].

Guatemala is the country comprising the highest number of individuals estimated to be living with HIV (49,000) in Central America, with prevalence in the general population of 0.5% [9]. The Guatemalan HIV epidemic is concentrated in most-at-risk groups, with 8.9% prevalence in men who have sex with men (MSM), 23.8% in transgender women and 1.0–2.6% in female sex workers (FSW) [10]. It is estimated that men who have sex with men comprise approximately 30% of the total estimated number of persons living with HIV in Guatemala and that heterosexual transmission accounts for 68% of cases [11]. The first AIDS case in Guatemala was recorded in 1984 in a young homosexual man who had resided in the United States, and cases in women were not reported until 1986 [12]. Geographic distribution of the Guatemalan HIV epidemic reflects economic development with the involvement of departments with higher commercial activity and with marked migratory routes, including Guatemala, Escuintla, San Marcos, Retalhuleu, Quetzaltenango, Izabal, Petén and Suchitepéquez, which concentrate 76% of notified cases [12]. A higher proportion of cases is found in Ladino (Mestizo) populations (77%) compared to Maya (indigenous) populations (20%), and 60% of infected individuals are male (approximately 60% of the general population is considered Ladino and 40% indigenous) [10]. Nevertheless, the molecular epidemiological history of HIV has not been assessed in Guatemala. For rapidly evolving pathogens such as HIV, phylogenetic methods can contribute to understand the complex dynamics of viral spread at the population level. Moreover, molecular epidemiology studies have the potential to better inform targeted public health interventions to contain the HIV epidemic at the local, national, or regional context, by identifying key clinical, demographic and geographical correlates of transmission. This information can help to focus limited resources to more effective HIV prevention efforts, including for example, the active search for new infection cases to link them to care and the roll out of pre-exposure prophylaxis [13].

The aim of the present study was to describe the spatiotemporal dynamics of dissemination of HIV subtype B in Guatemala, assessing its possible evolutionary relations with other viruses circulating in the Americas. The study was performed on a large HIV *pol* sequence dataset from individuals enrolled from 2010 to 2013 at the Roosevelt Hospital, one of the national HIV reference centers.

## Materials and methods

### Ethics statement

This study was evaluated and approved by the Ethics Committees of the National Institute of Respiratory Diseases (INER) in Mexico City (E06-09), and the Roosevelt Hospital in Guatemala City, and was conducted according to the principles of the Declaration of Helsinki. All participants gave written informed consent before blood sample donation.

### Guatemalan HIV-1 *pol* sequences

Blood samples from 1,083 HIV-1-infected Guatemalan individuals were collected at the Infectious Diseases Clinic of the Roosevelt Hospital in Guatemala City from October 2010 to December 2013 as part of an HIV pre-treatment drug resistance study [14]. Briefly, antiretroviral treatment-naïve individuals were enrolled in a cross-sectional study according to new clinical diagnoses at spontaneous demand to the clinic, also including individuals on follow-up visits, previous to starting antiretroviral treatment. Every eligible person attending the clinic was invited to participate in the study. No exclusion criteria were applied except for previous exposure to antiretroviral drugs. After giving written informed consent, participants donated a single blood sample. The complete protease (PR) and the first part of the reverse transcriptase (RT) of the pol gene (nucleotides 2253 to 3275 of reference strain HXB2) were amplified and sequenced using an in-house developed methodology as previously described [14]. Sequences used in the study are available at S1 Data. Serving 30% of all HIV-infected persons in Guatemala, the Roosevelt Hospital is the largest of a total of 18 clinics providing antiretroviral treatment in the country [15]. Although, the Roosevelt Hospital is located in the Department of Guatemala, the persons that initiate ART in this institution come from all over the country [16], with 45% of clients from other departments [14]. The place of residence distribution of Roosevelt Hospital’s clients reflects the national distribution of HIV cases [11, 16]. Also, sociodemographic characteristics (including female-to-male ratio, mean age, race, marital status and literacy) of Roosevelt Hospital’s clients is similar to those of the national HIV-infected population [16, 17].

### HIV-1 subtype B *pol* reference sequences

The HIV-1 subtype B *pol* sequences from Guatemala were aligned with: 1) subtype B *pol* sequences representative of the B_PANDEMIC_/B_CAR_ clades (US/France = 809/Caribbean = 238) [3, 8]; 2) Panamanian subtype B *pol* sequences representative of the country-specific clades (*n* = 583) [8]; and 3) all available subtype B *pol* sequences from other Central American countries (*n* = 694) and Mexico (*n* = 824) that matched the above selected genomic region (S1 Table). All HIV-1 subtype B *pol* reference sequences used in this study were available at Los Alamos HIV Sequence Database (www.hiv.lanl.gov) by December 2014.

### Sequence alignment and subtype assignment

Sequences were aligned using the ClustalW program implemented in Mega 6.0 software [18, 19]. The subtype assignment of all sequences included was confirmed using REGA HIV subtyping tool v.2 [20] and by performing Neighbor-Joining (NJ) phylogenetic analyses with HIV-1 group M reference sequences downloaded from Los Alamos HIV Sequence Database. The NJ phylogenies were constructed under the Tamura-Nei evolutionary model and the reliability of tree topologies was assessed by bootstrap analysis with 500 replicates using the MEGA 6.0 software package [19]. Bootstrap values above 75% were considered significant. All sites associated with major antiretroviral drug resistance mutations in PR (30, 32, 46, 47, 48, 50, 54, 76, 82, 84, 88 and 90) or RT (41, 65, 67, 69, 70, 74, 100, 101, 103, 106, 115, 138, 151, 181, 184, 188, 190, 210, 215, 219 and 230) detected in at least two sequences, were excluded from each dataset. All alignments are available from the authors upon request.

### Phylogenetic analysis

Maximum Likelihood (ML) phylogenetic trees were inferred under GTR+I+Γ_4_ nucleotide substitution model selected using the jModeltest program [21]. The ML tree was reconstructed with the PhyML program [22] using an online web server [23]. Heuristic tree search was performed using the SPR branch-swapping algorithm and the reliability of the obtained topology was estimated with the approximate likelihood-ratio test (*aLRT*) [24] based on the Shimodaira-Hasegawa-like procedure. *aLRT* values above 0.8 were considered significant. The ML trees were visualized using the FigTree v1.4.0 program [25].

### Analysis of spatiotemporal dispersion patterns

The evolutionary rate (nucleotide substitutions per site per year, subst./site/year), the age of the most recent common ancestor (*T*_mrca,_ years) and the spatial diffusion of HIV-1 Guatemalan clades were jointly estimated using the Bayesian Markov Chain Monte Carlo (MCMC) approach as implemented in BEAST v1.8 [26, 27] with BEAGLE [28]. Analyses were performed using the GTR+I+Γ_4_ nucleotide substitution model and the temporal scale of evolutionary process was directly estimated from the sampling dates of the sequences using a relaxed uncorrelated lognormal molecular clock model [29]. Migration events throughout the phylogenetic histories were reconstructed using a reversible discrete phylogeography model [30]. Adequate chain mixing and uncertainty in parameter estimates were assessed by calculating the effective sample size (ESS) and the 95% Highest Posterior Density (HPD) values respectively using the TRACER v1.5 program [31]. Maximum clade credibility (MCC) trees were summarized with TreeAnnotator v1.8.1 and visualized with FigTree v1.4.0. Migratory events were summarized using the SPREAD application [32].

### Reconstruction of demographic history

The mode and rate (*r*, years^-1^) of population growth of major HIV-1 clades circulating in Guatemala was also estimated using BEAST v1.8 software. Analyses were also performed using the GTR+I+Γ_4_ nucleotide substitution model and the temporal scale of evolutionary process were estimated using a relaxed uncorrelated lognormal molecular clock model. Changes in effective population size through time were initially estimated using a Bayesian Skyline coalescent tree prior [33] and estimates of the population growth rate were subsequently obtained using the parametric model (logistic, exponential or expansion) that provided the best fit to the demographic signal contained in datasets. Comparison between demographic models was performed using the log marginal likelihood (ML) estimation based on path sampling (PS) and stepping-stone sampling (SS) methods [34]. MCMC chains were run for 50–500 × 10^6^ generations. Convergence and uncertainty of parameter estimates were assessed as explained above.

### Statistical analysis

Demographic and clinical variables of individuals belonging to the different clades were compared using linear regression or Chi-square test according to variable type. False Discovery Rate (FDR) for multiple comparisons was estimated with Storey q-values. The threshold for significance was set at p<0.05 and q<0.2. Analysis was performed using STATA/SE version 14 (StataCorp, College Station, TX).

## Results

### Most HIV-1 subtype B Guatemalan sequences belong to the B_PANDEMIC_ clade

**Fig 1:**
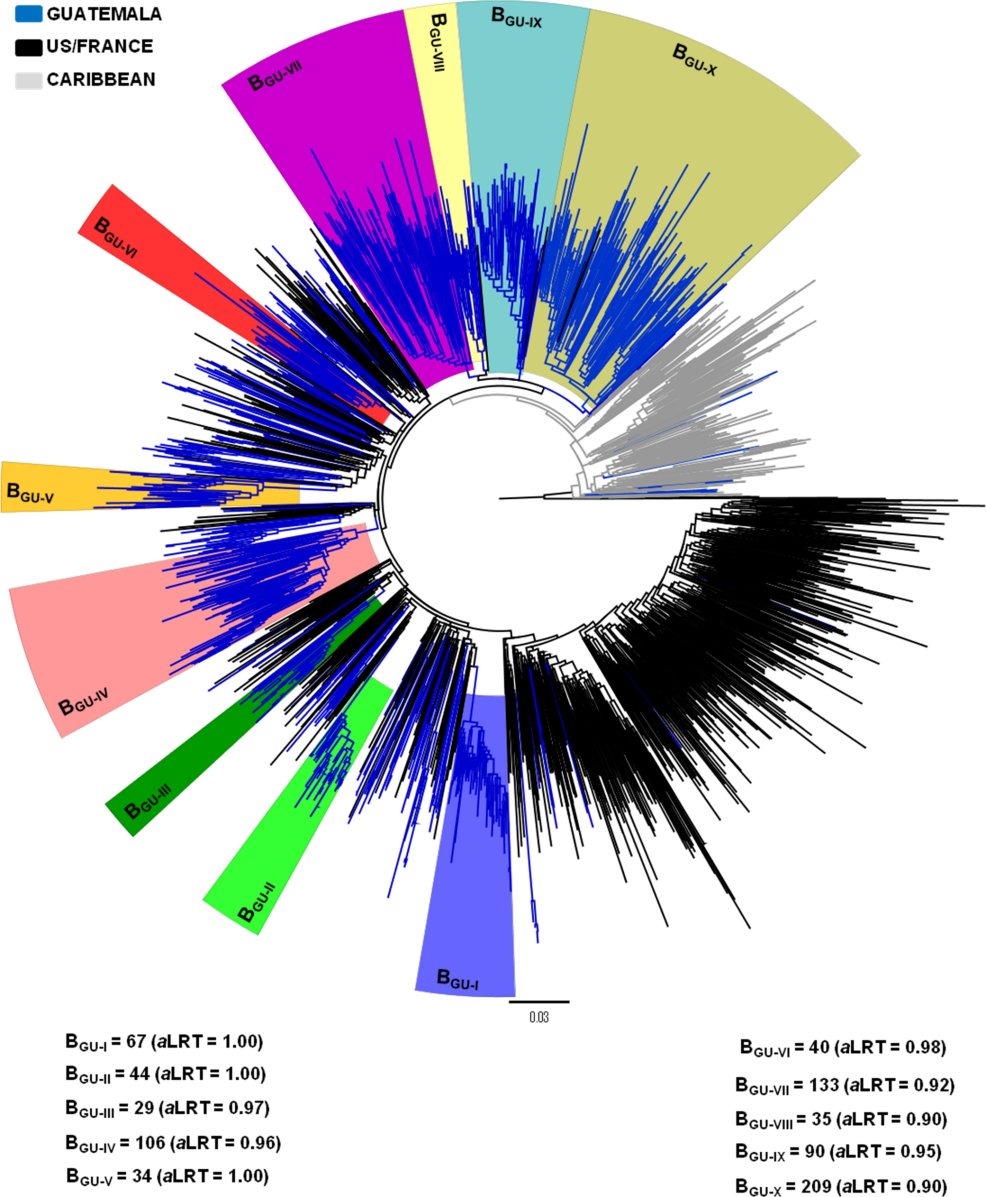
Maximum likelihood phylogenetic tree of HIV-1 subtype B *pol* (~1000 pb) sequences circulating in Guatemala (*n* = 1047), and representative sequences of the B_PANDEMIC_ (US = 465, France = 344) and the B_CAR_ (Caribbean = 238) clades. Branches are colored according to the geographic origin of each sequence, as indicated in the legend (top left). Colored boxes highlight the position of the ten major Guatemalan HIV-1 subtype B clades (B_GU-I_ to B_GU-X_). The number of sequences and the *a*LRT support values for each clade are indicated at the bottom. The tree was rooted using HIV-1 subtype D reference sequences. Branch lengths are drawn to scale with the bar at the bottom indicating nucleotide substitutions per site.

Of the 1,083 HIV-1 *pol* Guatemalan sequences here analyzed, we selected 1,047 subtype B sequences geographically distributed among the 22 departments of Guatemala. The remaining 36 sequences were excluded because of possible quality issues (unusual indel mutations/rare polymorphisms) (*n* = 21) or non-B subtype classification (*n* = 15). In order to better understand the origin of the Guatemalan HIV-1 subtype B epidemic, Guatemalan sequences were combined with representative sequences of the B_PANDEMIC_ and B_CAR_ lineages (S1 Table) to perform a phylogenetic analysis. The ML analysis revealed that, as expected, the B_CAR_ sequences occupied the deepest branches within the subtype B phylogeny; whereas the B_PANDEMIC_ sequences branched as a well-supported (*aLRT* = 0.88) monophyletic subgroup nested within the B_CAR_ clades (Fig 1). Of the 1,047 sequences analyzed from Guatemala, 1,036 (98.9%) branched within the B_PANDEMIC_ clade and only 11 sequences (1.1%) branched among the B_CAR_ lineages (Fig 1). This analysis also revealed that 787 (75.2%) of the Guatemalan sequences branched in 10 country-specific monophyletic clades of large size (29 ≤ *n* ≤ 209; B_GU-I_ to B_GU-X_), 226 (21.6%) branched in 43 country-specific clusters of small size (2 ≤ *n* ≤ 17), and the remaining 34 (3.2%) represented non-clustered sequences (Fig 1).

### Identification of country- and region-specific HIV-1 subtype B clades

**Fig 2:**
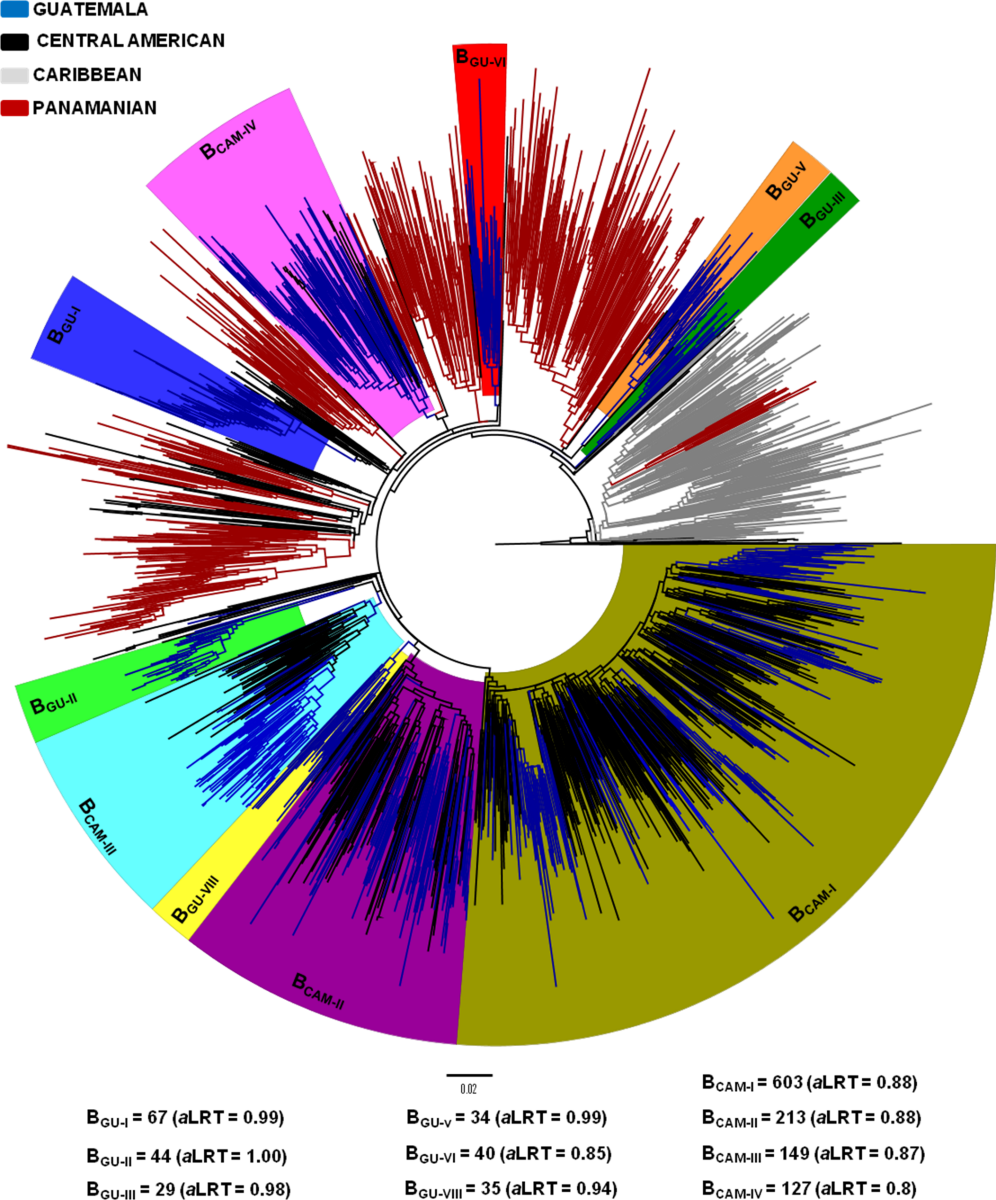
Maximum likelihood phylogenetic tree of HIV-1 subtype B *pol* (~1000 pb) sequences belonging to the major Guatemalan clades (B_GU-I_ to B_GU-X,_ n = 787), and reference sequences from various Central American countries (*n* = 694), Panama (*n* = 583), and the Caribbean (*n* = 238). Branches are colored according to the geographic origin of each sequence, as indicated in the legend (top left). Colored boxes highlight the position of the six major Guatemalan HIV-1 subtype B clades (B_GUs_) and the four Central American clades (B_CAM-I_ to B_CAM-VI_). The number of sequences and *a*LRT support values for each clade are indicated at the bottom. The tree was rooted using HIV-1 subtype D reference sequences. Branch lengths are drawn to scale with the bar at the bottom indicating nucleotide substitutions per site.

**Fig 3:**
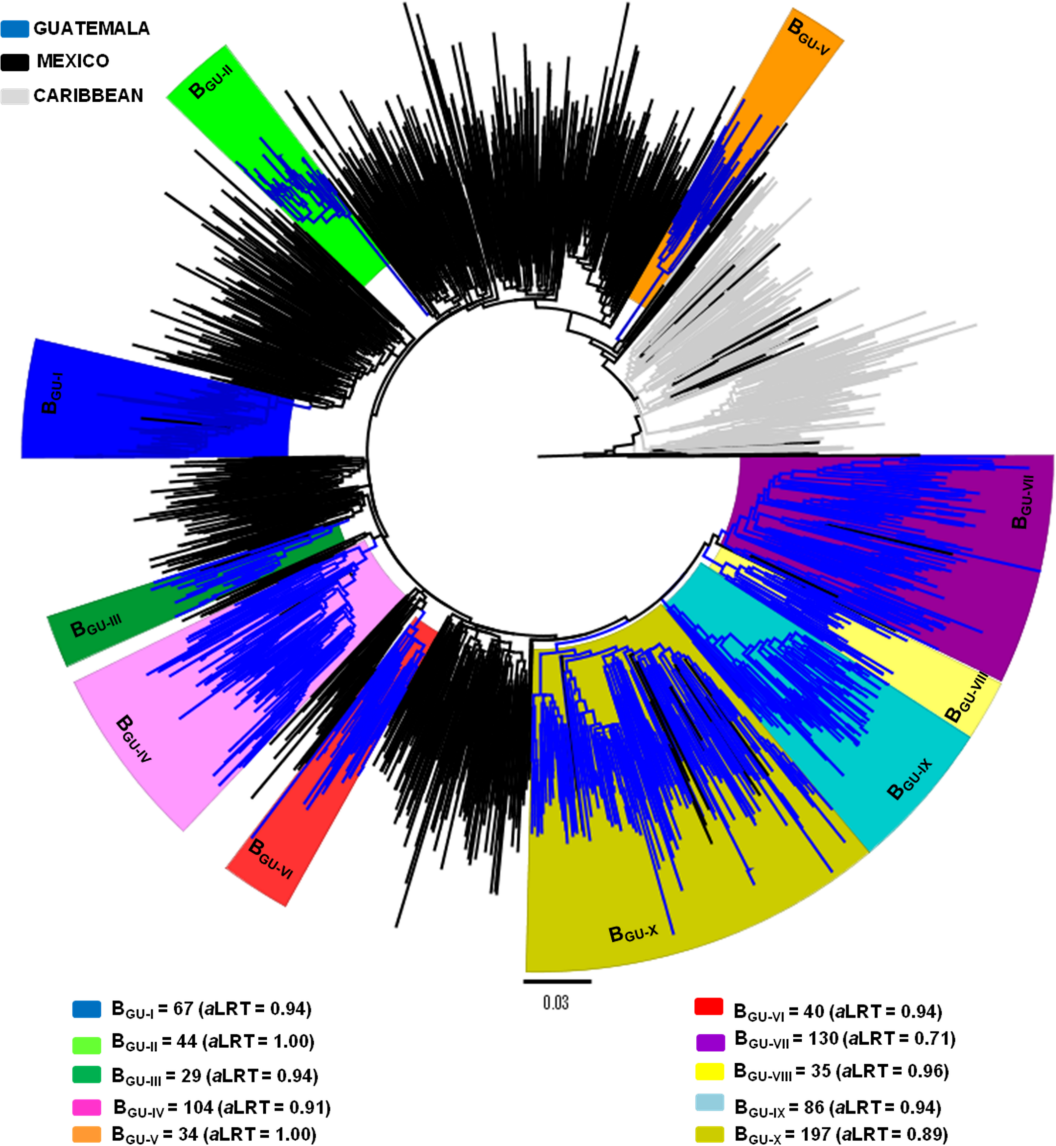
Maximum likelihood phylogenetic tree of HIV-1 subtype B *pol* (~1000 bp) sequences belonging to the major Guatemalan clades (B_GU-I_ to B_GU-X,_ n = 787), and reference sequences from Mexico (*n* = 824) and the Caribbean (*n* = 238). Branches are colored according to the geographic origin of each sequence, as indicated in the legend (top left). Branches from clades B_GU-I_ to B_GU-X_ are colored according to clade as indicated in the legend (bottom). The tree was rooted using HIV-1 subtype D reference sequences. Branch lengths are drawn to scale with the bar at the bottom indicating nucleotide substitutions per site.

HIV-1 subtype B sequences from the largest Guatemalan clades here identified were next aligned with subtype B sequences from Mexico, Panama and other Central American countries (S1 Table). ML analysis revealed that all sequences from Panama and Costa Rica and most sequences from Mexico (98%) branched outside the major Guatemalan clades (Figs 2 and 3). By contrast, a large proportion of sequences from Honduras (92%), El Salvador (54%), and Belize (22%) branched within the previously defined Guatemalan clades B_GU-IV_, B_GU-VII_, B_GU-IX_ and B_GU-X_ that were thus reclassified as Central American clades B_CAM-IV_, B_CAM-II_, B_CAM-III_ and B_CAM-I_, respectively (Fig 2). The prevalence of each major Central American clade greatly varied among countries (S2 Table). In Guatemala, the predominant clade was B_CAM-I_ (19%), followed by clades B_CAM-II_ (12%), B_CAM-IV_ (10%) and B_CAM-III_ (8%). In Honduras, the most prevalent clade was B_CAM-I_ (73%), followed by clades B_CAM-III_ (12%) and B_CAM-II_ (7%). In El Salvador, the predominant clade was B_CAM-II_ (34%), followed by clades B_CAM-IV_ (12%) and B_CAM-I_ (8%). In Belize, B_CAM-I_ and B_CAM-II_ clades seemed to circulate with similar prevalence (11%). Guatemalan clades (B_GU-I_, B_GU-II_, B_GU-III,_ B_GU-V_, B_GU-VI_ and B_GU-VIII_) were mostly (>99%) composed by sequences from that country and were thus classified as country-specific clades. Among subtype B sequences from Guatemala included in the study (*n* = 1,047), 49.3% (*n* = 516) belonged to Central America-specific clades and 23.8% (*n* = 249) belonged to Guatemala-specific clades.

### Epidemiological characteristics of Guatemalan subjects across major HIV-1 subtype B clades circulating in Central America

We then analyzed the demographic and clinical characteristics of individuals included in the different Guatemalan and Central American clades (Table 1). Significant differences in all the variables analyzed were observed between the B_GU-II_ clade and the other B_CAM_ and B_GU_ lineages. The B_GU-II_ clade was mostly transmitted among young (median age 27 years), recently infected (59%), MSM (46%), with higher education level (71% with bachelor degree and 16% with high-school degree) and employment (64%) rates. In sharp contrast, all other B_CAM_ and B_GU_ clades were mostly composed of older (median age >31 years), heterosexual (>79%) individuals, with long-standing infection (>78%), and lower education (degree <21%) and employment (<53%) rates than those observed in individuals infected by the B_GU-II_ clade. Guatemala City was the residence for most (>48%) individuals of major B_CAM_ and B_GU_ clades, with exception of B_GU-V_ (24%) and B_CAM-III_ (34%). The B_GU-V_ clade stands out due to a lower mean CD4 T cell count. This clade was composed by persons from outside the Department of Guatemala (77%), with a significantly lower level of education (35% with none and 53% with elementary degree) and heterosexual risk of transmission. Late arrival to clinical care in populations with lower education and socio-economic status is a common feature of many Latin American countries [35]. This may explain the common feature of low CD4 T cell counts at the time of HIV diagnosis.

**Table 1:** Clinical and demographic characteristics of individuals included in the large Guatemalan and Central American clusters.

	All clusters	B GU-I	B GU-II	B GU-III	B GU-V	B GU-VI	B GU-VIII	B CAM I	B CAM II	B CAM III	B CAM IV
**n**	765	67	44	29	34	40	35	197	129	86	104
**%**	100	8.8	5.8	3.8	4.4	5.2	4.6	25.8	16.9	11.2	13.6
**Age (years)**											
Median	34	37	**27**	**40**	31	36	36	35	35	33	34
IQR	28–44	29–46	**22–30**	**31–51**	28–42	29–43	29–48	30–44	29–45	27–44	29–43
**Gender (%)**											
Female	43.9	52.2	**6.8**	44.8	44.1	52.5	**65.7**	45.2	45	46.5	37.5
Male	56.1	47.8	**93.2**	55.2	55.9	47.5	**34.3**	54.8	55	53.5	62.5
**Civil Status (%)**											
Single	38.8	29.9	**81.8**	55.2	29.4	32.5	42.9	35	**31**	**38.4**	43.3
Married	24.2	**34.3**	**9.1**	24.1	**38.2**	22.5	22.9	**18.3**	29.5	23.3	26
Domestic Partnership	27.6	26.9	**6.8**	13.8	26.5	**35**	28.6	35	31.8	25.6	20.2
Divorced	1.31	1.49	0	0	0	2.5	0	1.52	0.78	1.16	2.88
Widow	8.1	7.5	2.3	6.9	5.9	7.5	5.7	10.2	7	11.6	7.7
**Education (%)**											
None	19	26.9	**4.6**	24.1	**35.3**	22.5	22.9	15.2	24.8	17.4	**11.5**
Elementary	50.6	56.7	**9.1**	51.7	52.9	52.5	54.3	54.3	52.7	53.5	49
High-school	15.6	9	15.9	3.4	8.8	12.5	20	16.2	13.2	**24.4**	19.2
Degree	14.9	7.5	**70.5**	20.7	**2.9**	12.5	**2.9**	14.2	**9.3**	**4.7**	20.2
**Employment (%)**											
Employed	46	49.3	**63.6**	51.7	52.9	37.5	34.3	42.6	50.4	43	43.3
Unemployed	54	50.7	**36.4**	48.3	47.1	62.5	65.7	57.4	49.6	57	56.7
**Recency of infection (%)**											
Recent	18.3	13.4	**59.1**	6.9	8.8	7.5	20	16.2	15.5	22.1	18.3
Long-standing	81.7	86.6	**40.9**	93.1	91.2	92.5	80	83.8	84.5	77.9	81.7
**HIV risk factor (%)**											
Heterosexual	87.6	**97**	**38.6**	86.2	94.1	90	94.3	**93.4**	90.7	91.9	**78.9**
MSM	8.2	**0**	**45.5**	6.9	2.9	5	2.9	**3.6**	7.8	**2.3**	**17.3**
Bisexual	3.3	1.5	15.9	6.9	0	5	2.9	2	1.6	3.5	2.9
PWID	0.5	1.5	0	0	0	0	0	0.5	0	1.2	1
Other/Unknown	0.4	0	0	0	**2.9**	0	0	0.5	0	1.2	0
**Residence (%)**											
Guatemala City	53.1	49.3	**77.3**	**79.3**	**23.5**	52.5	48.6	55.3	48.1	**33.7**	**67.3**
Not Guatemala City	46.9	50.7	**22.7**	**20.7**	**76.5**	47.5	51.4	44.7	51.9	**66.3**	**32.7**
**Viral Load (log copies/mL)**											
Mean	4.86	**5.1**	4.89	4.96	4.82	4.87	4.86	4.85	4.74	**5.09**	**4.67**
IQR	4.34–5.37	**4.47–5.62**	4.44–5.26	4.59–5.47	4.47–5.18	4.39–5.33	4.16–5.38	4.23–5.38	4.24–5.15	**4.44–5.53**	**4.10–5.13**
**CD4+ T cell count (cells/μL)**											
Mean	167	146	**399**	157	**80**	142	214	156	139	172	178
IQR	66–346	81–327	**256–481**	62–330	**29–206**	52–297	90–362	61–353	66–309	65–292	82–388

Significant values (each cluster vs. all the other clusters; p<0.5, q<0.2) are shown in bold.

### Spatiotemporal dissemination of major HIV-1 subtype B clades circulating in Central America

**Fig 4:**
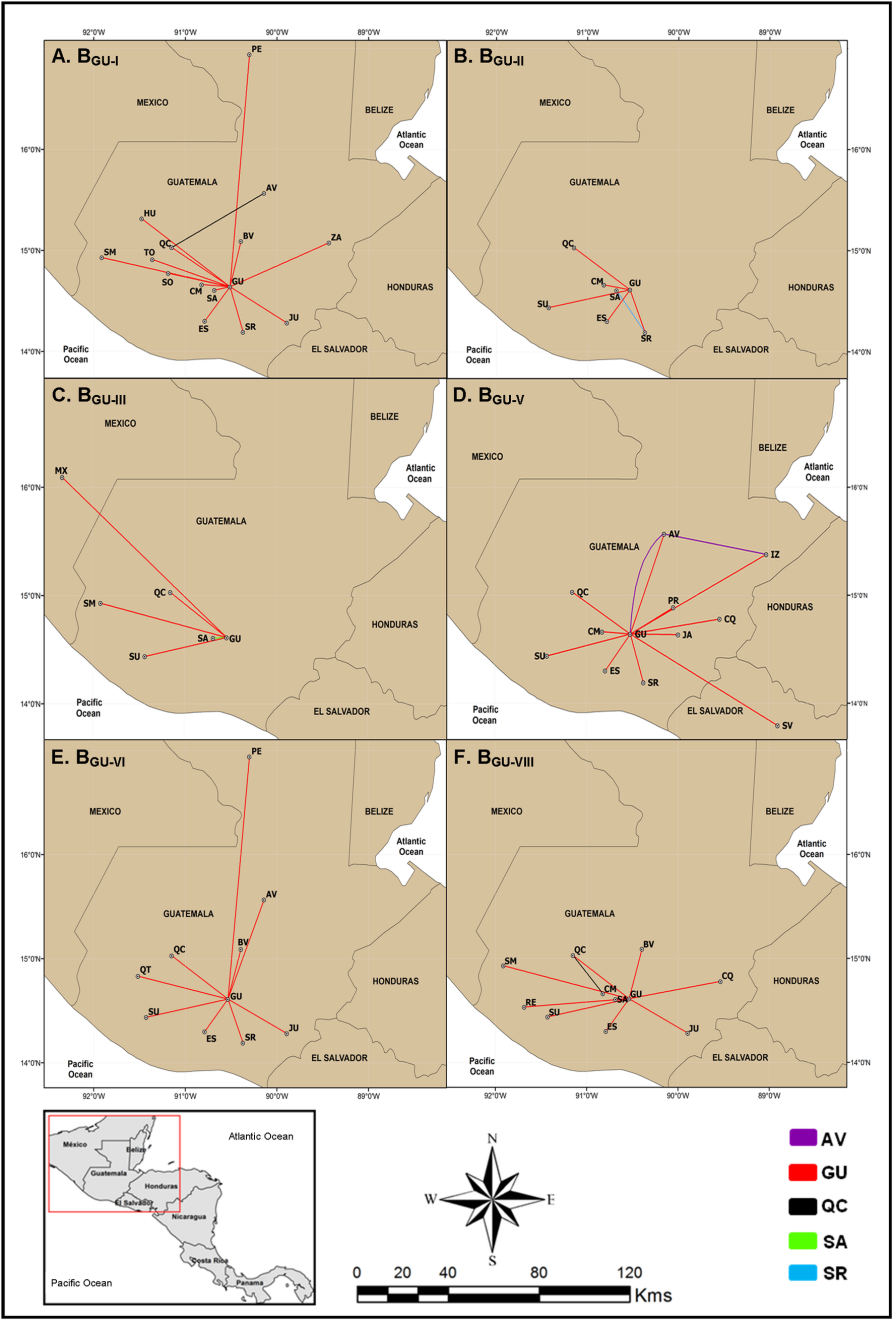
Spatial dissemination of HIV-1 country-specific Guatemalan clades (B_GU_). Lines between departments and neighboring countries represent branches in the Bayesian MCC tree along which location transitions occur. Lines were colored according to the source location as indicated in the legend at the bottom. Dashed lines indicate transitions associated to nodes with low location probability support (<0.60). Guatemalan Departments: Alta Verapaz (AV), Baja Verapaz (BV), Chimaltenango (CM), Chiquimula (CQ), El Progreso (PR), Escuintla (ES), Guatemala (GU), Huehuelenango (HU), IZABAL (IZ), Jalapa (JA), Jutiapa (JU), PETEN (PE), Quetzaltenango (QT), Quiche (QC), Retalhuleu (RE), Sacatepequez (SA), San Marcos (SM), Santa Rosa (SR), Solola (SO), Suchitepequez (SU), Totonicapan (TO) and Zacapa (ZA).

The median estimated evolutionary rates at HIV-1 *pol* gene for major Guatemalan and Central American HIV-1 clades ranged from 2.1 x 10^−3^ subst./site/year to 2.9 x 10^−3^ subst./site/year with a great overlap of 95% HPD intervals, whereas the coefficient of variation ranged from 0.33 (0.14–0.50) to 0.55 (0.34–0.76), thus supporting the use of a relaxed molecular clock model (Table 2). The HIV-1 subtype B Guatemalan clades B_GU-III_, B_GU-V_, B_GU-VI_ and B_GU-VIII_ arose between the early and the middle 1990s, followed by clades B_GU-I_ and B_GU-II_ between the early and the middle 2000s (Table 2). The phylogeographic analysis showed that the department of Guatemala was the most probable epicenter of dissemination of all those Guatemalan clades (*PSP*≥0.86). B_GU-I_ was the most widely disseminated lineage, being detected in 15 out of the 22 Guatemalan departments, followed by clade B_GU-V_ detected in 11 departments and El Salvador; clade B_GU-VIII_ detected in 11 departments, clade B_GU-VI_ in 10 departments; clade B_GU-II_ in seven departments, and clade B_GU-III_ in six departments and Mexico (Fig 4). The departments of Alta Verapaz, Quiche, Sacatepequez and Santa Rosa were also detected as secondary disseminating locations of clades B_GU-V_, B_GU-I_/B_GU-II_/B_GU-VIII_, B_GU-III_ and B_GU-II_, respectively (Fig 4).

**Table 2:** Evolutionary rate and time-scale estimates of B_GU_ and B_CAM_ clades.

Clade	*N*	Sampling interval	*μ* (subst./site/year)^a^	Variation Coefficient^a^	T_*MRCA*_^a^
B_GU-I_	67	2010–2013	2.2×10^−3^ (1.5×10^−3^–2.9×10^−3^)	0.55 (0.34–0.76)	2000 (1994–2004)
B_GU-II_	44	2010–2013	2.4×10^−3^ (1.7×10^−3^–3.0×10^−3^)	0.37 (0.004–0.65)	2005 (2002–2007)
B_GU-III_	29	2010–2013	2.1×10^−3^ (1.5×10^−3^–2.8×10^−3^)	0.42 (0.07–0.75)	1991 (1980–2000)
B_GU-V_	34	2010–2013	2.0×10^−3^ (1.5×10^−3^–2.8×10^−3^)	0.43 (0.23–0.65)	1992 (1983–2000)
B_GU-VI_	40	2010–2013	2.2×10^−3^ (1.5×10^−3^–2.9×10^−3^)	0.33 (0.14–0.50)	1994 (1986–2001)
B_GU-VIII_	35	2010–2013	2.2×10^−3^ (1.5×10^−3^–2.9×10^−3^)	0.46 (0.24–0.68)	1994 (1986–2001)
B_CAM-I_	509	2001–2013	2.9 × 10^−3^ (2.8×10^−3^–3.0×10^−3^)	0.33 (0.29–0.37)	1987 (1984–1989)
B_CAM-II_	224	2003–2013	2.3 × 10^−3^ (1.7×10^−3^–2.9×10^−3^)	0.21 (0.14–0.28)	1978 (1968–1986)
B_CAM-III_	150	2001–2013	2.9 × 10^−3^ (2.7×10^−3^–3.0×10^−3^)	0.31 (0.22–0.41)	1987 (1984–1990)
B_CAM-IV_	126	2005–2013	1.9×10^−3^ (1.5×10^−3^–2.6×10^−3^)	0.30 (0.22–0.39)	1980 (1971–1990)

^a^95% Higher Posterior Density intervals are shown in parentheses.

**Fig 5:**
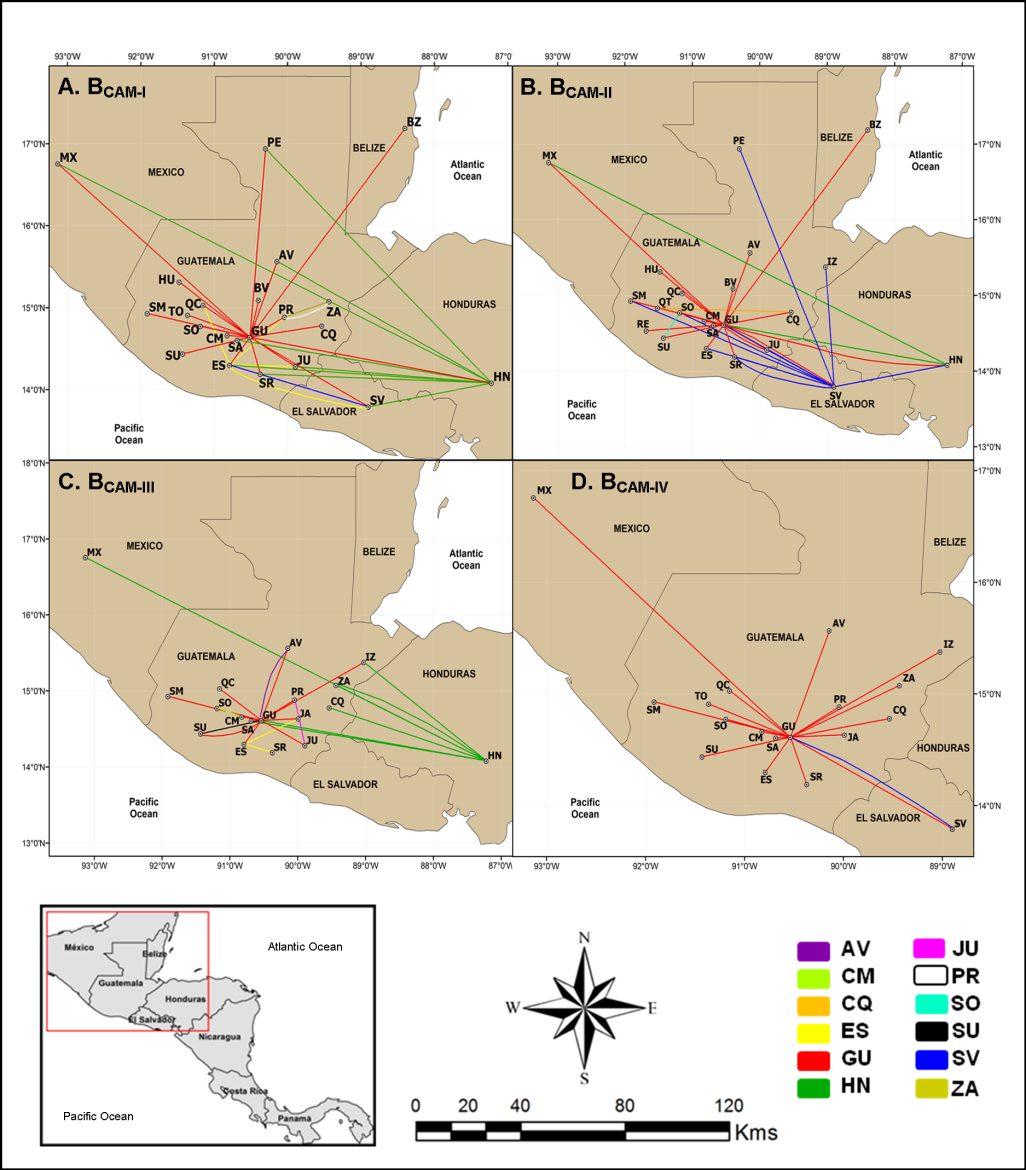
Spatial dissemination of HIV-1 Central American clades circulating in Guatemala (B_CAM-I_ to B_CAM-IV_). Lines between departments and neighboring countries represent branches in the Bayesian MCC tree along which location transitions occur. Lines were colored according to the source location as indicated in the legend at the bottom. Dashed lines indicate transitions associated to nodes with low location probability support (<0.60). Countries: Belize (BZ), El Salvador (SV), Mexico (MX) and Honduras (HN). Guatemalan Departments: Alta Verapaz (AV), Baja Verapaz (BV), Chimaltenango (CM), Chiquimula (CQ), El Progreso (PR), Escuintla (ES), Guatemala (GU), Huehuelenango (HU), IZABAL (IZ), Jalapa (JA), Jutiapa (JU), PETEN (PE), Quetzaltenango (QT), Quiche (QC), Retalhuleu (RE), Sacatepequez (SA), San Marcos (SM), Santa Rosa (SR), Solola (SO), Suchitepequez (SU), Totonicapan (TO) and Zacapa (ZA).

The root location of Central American clades B_CAM-I_ and B_CAM-III_ was most probably traced to Honduras (Posterior State Probability, *PSP* ≥0.99) around the middle 1980s (Table 2). The most widely disseminated viral clade, B_CAM-I,_ migrated from Honduras to the department of Guatemala, El Salvador and Mexico during the early 1990s and later moved to other Guatemalan departments around 2010 (Fig 5). From the department of Guatemala, clade B_CAM-I_ was disseminated to other 16 Guatemalan departments and also to Mexico, Belize, and back to Honduras. El Salvador and the Guatemalan department of Escuintla were also secondary hubs of dissemination of clade B_CAM-I_. Clade B_CAM-III_ moved from Honduras to several Guatemalan departments (Guatemala, Zacapa, Sacatepéquez, Izabal and Chiquimula) and to Mexico since 1997 (Fig 5). The department of Guatemala acted as a secondary hub of dissemination of B_CAM-III_ sending viruses to other Guatemalan departments from 2001 onwards.

The root location for clades B_CAM-II_ and B_CAM-IV_ was most probably traced to the department of Guatemala (*PSP* = 0.47 and 0.99, respectively) between the late 1970s and the early 1990s (Table 2). Clade B_CAM-II_ moved from the department of Guatemala to Honduras and El Salvador around the late 1970s, to the Guatemalan departments of Solola and Chiquimula during the 1990s, and to other 11 Guatemalan departments, Belize and Mexico since the early 2000 (Fig 5). Secondary hubs of dissemination of clade B_CAM-II_ were El Salvador from where the virus has spread to Honduras, back to the department of Guatemala, and to other seven Guatemalan departments since the early 1990s, and Honduras from where the virus has spread back to the department of Guatemala and Mexico since the early 1980s. The department of Guatemala was the major epicenter of clade B_CAM-IV_ moving from the capital to other 15 Guatemalan departments, as well as to El Salvador and Mexico since the middle 1980s. From El Salvador, clade B_CAM-IV_ moved back to the department of Guatemala.

### Demographic history of major HIV-1 subtype B clades circulating in Guatemala

**Fig 6:**
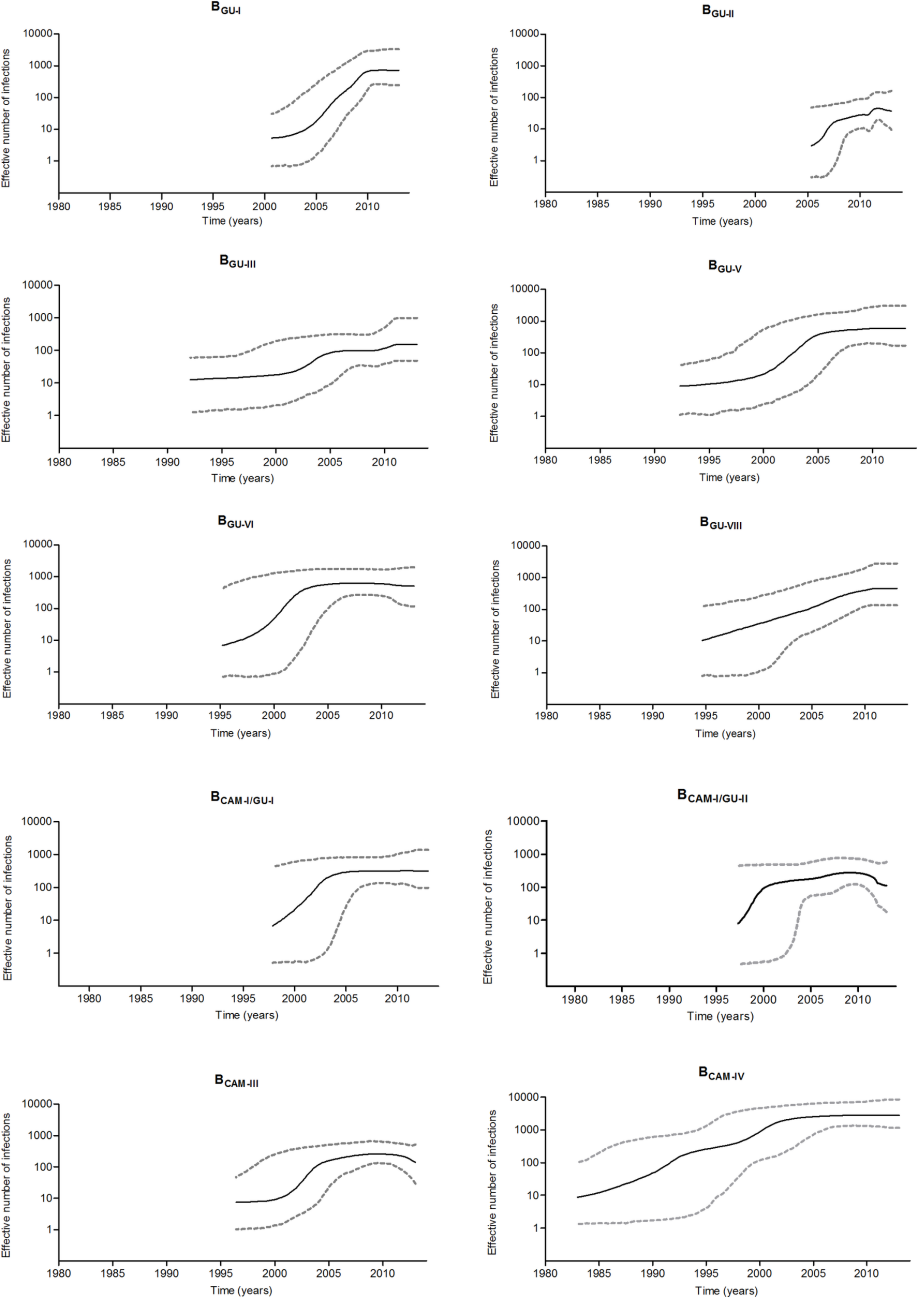
Demographic history of HIV-1 Guatemalan clades. Effective number of infections per year was estimated using a Bayesian skyline growth coalescent model for each one of the Guatemalan clades. Median estimates of the effective number of infections (solid line) and 95% High Posterior Density intervals of the estimates (dashed lines) are shown.

To reconstruct the population dynamics of the HIV-1 subtype B epidemic in Guatemala, we used the Guatemalan sequences from those clades that arose in Guatemala (B_GU-I_, B_GU-II_, B_GU-III_, B_GU-V_, B_GU-VI_ and B_GU-VIII_, and B_CAM-IV_) and well supported (aLTR ≥80) Guatemala-specific sub-clades within clades B_CAM-I_ (B_CAM-I/GU-I_ = 51 sequences and B_CAM-I/GU-II_ = 37) and B_CAM-III_ (B_CAM-III/GU_ = 71 sequences). Bayesian skyline plot (BSP) analyses suggest that most clades experienced an initial phase of exponential growth followed by a decline in growth rate during the 2000s, leading to stabilization of the effective population size (Ne) (Fig 6). Some clades (B_GU-II_, B_GU-III_, B_CAM-IV_ and B_CAM-I/GU-II_), however, seem to have experienced more complex dynamics characterized by phases of expansion interleaved by periods of slow growth (Fig 6). Model comparison indicated that the best-fit demographic model was the logistic for clades B_GU-II,_ B_CAM-IV,_ B_GU-VI_, B_CAM-I/GU-I_ and B_CAM-I/GU-II_, while both the logistic and exponential models fit equally well the demographic signal in clades B_GU-I,_ B_GU-III,_ B_GU-V_, B_GU-VIII_ and B_GU-IX_ (S3 Table). According to these parametric models, the mean estimated growth rate of major clades circulating in Guatemala ranged from 0.23 year^-1^ for clade B_GU-III_ to 3.59 year^-1^ for clade B_GU-II_ (Table 3).

**Table 3:** Population dynamics estimates for Guatemalan HIV-1 subtype B clades.

Clade	Demographic model	*T*_MRCA_^a^	Growth rate (yr^-1^)^a^
B_GU-I_	Exponential	2000 (1995–2004)	0.58 (0.36–0.82)
	Logistic	2000 (1996–2005)	0.70 (0.39–1.11)
B_GU-II_	Logistic	2005 (2002–2007)	3.59 (0.30–9.02)
	Exponential	2004 (2000–2007)	0.41 (0.19–0.66)
B_GU-III_	Exponential	1993 (1983–2001)	0.24 (0.10–0.42)
	Logistic	1992 (1982–2001)	0.23 (0.00–2.34)
B_GU-V_	Exponential	1993 (1985–2000)	0.33 (0.21–0.52)
	Logistic	1993 (1986–2000)	0.43 (0.21–0.76)
B_GU-VI_	Logistic	1996 (1996–2001)	0.99 (0.48–1.72)
B_GU-VIII_	Exponential	1995 (1987–2001)	0.36 (0.19–0.54)
	Logistic	1995 (1987–2001)	0.38 (0.13–0.68)
B_CAM-I/GU-A_	Logistic	1998 (1993–2003)	1.46 (0.52–3.00)
B_CAM-I/GU-B_	Logistic	1997 (1991–2002)	2.09 (0.61–5.03)
B_CAM-III/GU_	Logistic	1997 (1990–2001)	0.68 (0.35–1.12)
B_CAM-IV/GU_	Logistic	1984 (1975–1994)	0.40 (0.26–0.63)

^a^95% Higher Posterior Density intervals are shown in parentheses.

## Discussion

The HIV-1 subtype B epidemic in Guatemala is mostly related to the B_PANDEMIC_ clade (98.9%), as described for the majority of the Central [7, 8, 36], North, and South [3, 37] American countries. The proportion of Guatemalan HIV-1 subtype B viruses belonging to the B_CAR_ clades (1.1%) was similar to the prevalence reported for Honduras, El Salvador, and Mexico (<1.1%) [37], but lower than the one reported in Panama (5.5%), the country where B_CAR_ clade circulation was first demonstrated in Central America [8].

Our phylogeographic analysis identified both country- and region-specific HIV-1 subtype B clades involved in the Guatemalan HIV epidemic, contrasting with the Panamanian epidemic characterized mostly by the dissemination of country-specific clades [8]http://www.ncbi.nlm.nih.gov/pubmed/?term=Uribe-Salas%20F%5BAuthor%5D&cauthor=true&cauthor_uid=12616149. Our study further reveals the existence of at least four major regional-specific clades in Central America (BCAM-I—B_CAM-IV_), contrasting with the previous observation of a single HIV subtype B linage (B_CAM_ clade) accounting for most of the HIV epidemic burden in Central America [7]. The fact that the Central American clades BCAM-I—B_CAM-IV_ differ in root location (B_CAM-I_ and B_CAM-III_ traced to Honduras, and B_CAM-II_ and B_CAM-IV_ traced to Guatemala), also refute the Murillo *et al* assertion that a single viral introduction event was responsible for most of the HIV-1 subtype B infections in Central America. This change in the previously described HIV-1 epidemiological history of subtype B in Central America is expected, considering the fact that the highest number of individuals estimated to be living with HIV in Central America are from Guatemala [9], and this country was not included in previous analyses.

Nearly half of all Guatemalan HIV-1 subtype B sequences included in our study branched within Central American B_PANDEMIC_ clades (67%), indicating an unrestricted viral flow between main transmission networks from Guatemala and the neighboring Central American countries of Honduras and El Salvador. This result is consistent with a previous study that suggested an HIV-1 subtype B epidemiological link between the Guatemalan neighboring countries Belize, Honduras and El Salvador [6]. A much more restricted viral flow, by contrast, was observed between Guatemala and Mexico (despite their close geographic proximity) and between Guatemala and Panama. Viral flux between Guatemala and Belize, Nicaragua and Costa Rica was not possible to estimate because of the paucity of HIV-1 *pol* sequences from these countries.

About one fourth of all Guatemalan HIV-1 subtype B sequences included in our study branched within six country-specific clades of large size (B_GU-I_, B_GU-II_, B_GU-III,_ B_GU-V_, B_GU-VI_, and B_GU-VIII_). The analysis of clinical and demographic characteristics, however, reveals no major difference between B_CAM_ and B_GU_ clades, with the exception of clade B_GU-II_ that differed from all other clades mostly comprising mainly young, recently infected MSM with higher education and employment rates. In the last 10 years, HIV prevalence among MSM has remained higher than 8% in Guatemala, with most of the cases (75%) related to MSM younger than 36 years with higher education [38]. Major differences, by contrast, were detected between B_CAM_ and B_GU_ clades, with regards to the estimated origin of the different lineages: B_GU_ clades displayed a much more recent median T_MRCA_ (between the 1990s and 2000s) than the B_CAM_ clades (between the 1970s and 1980s), which may have had a great impact in the geographical expansion of each lineage.

The spatiotemporal dissemination analysis of B_GU_ and B_CAM_ clades evidenced complex migration patterns characterizing the HIV-1 epidemic in the region, involving Guatemalan departments and the neighboring countries Honduras, El Salvador, Belize and Mexico. Guatemalan internal and external migration has been attributed mostly to economic factors, with population mobility from poor rural areas to agriculture or industrial developing areas [39], and the country’s civil war that lasted 32 years, from 1960 to 1996 [40]. The department of Guatemala and, to a lesser extent, Escuintla, Izabal and Petén have been the main destinations for internal-migration from the eastern and western departments of the country, with dynamic bidirectional population mobility [41, 42]. Regarding external migration, 50% of immigrants to Guatemala correspond to people of other Central American countries, mostly from El Salvador, Nicaragua and Honduras [42], yet the Guatemalan net migration rate is negative [43] with emigration flows mostly to the United States of America, and the neighboring countries of Mexico, Honduras, El Salvador and Belize [42]. Our phylogeographic reconstructions revealed that the department of Guatemala was the dissemination epicenter of the B_GU_, B_CAM-II_ and B_CAM-IV_ clades to the rest of Guatemalan departments and to the Guatemalan neighboring countries. Additionally, the departments of Guatemala and Escuintla served as secondary hubs of dissemination of the B_CAM-I_ and B_CAM-III_ clades, which emerged in Honduras, to other Guatemalan departments and to the four bordering countries in the case of B_CAM-I_. The estimated T_MRCA_ of B_GU_ clades, ranging between the early 1990s and the middle 2000s, also coincides with an increased repatriation and deportation rate of Guatemalans from Mexico and the United States of America, respectively [42, 44], although we found no significant evolutionary links between B_GU_ clades and the Mexican subtype B epidemic.

The demographic analysis of the B_GU-I_, B_GU-V_, B_GU-VI_, B_GU-VIII_, and B_CAM-I/GU-I_ clusters suggested that, even though all these clades had an initial phase of exponential growth, the dissemination of these clades declined during the 2000s. On the other hand, the B_GU-II_, B_GU-III_, B_CAM-IV_ and B_CAM-I/GU-II_ clades evidenced a more complex growth dynamics with phases of expansion interleaved by periods of slow growth. The B_GU-II_ clade, characterized mostly by a population of young MSM, evidenced the highest mean estimated logistic growth rate (3.6 year^-1^) among the major clades circulating in Guatemala. The B_GU-II_ clade growth rate was remarkably higher than those previously estimated for other country-specific B clades mainly or exclusively restricted to MSM (ranging from 0.5 to 1.6 year^-1^) [45–48]. Furthermore, the proportion of strains that corresponds to new infections within the B_GU-II_ clade was higher (59.1%) compared with the rest of the B_GU_ and B_CAM_ clades (ranging from 6.9% of B_GU-III_ to 22.1% of B_CAM-III_). As the HIV epidemic in Guatemala is concentrated in high-risk key populations, with 8.9% prevalence in MSM [10], an increase in size and proportion of the B_GU-II_ clade would be expected in the current Guatemalan HIV epidemic. These results, however, should be interpreted with caution because the exponential growth model, that was equally supported as the logistic one, showed a much lower mean growth rate (0.4 year^-1^) for the B_GU-II_ clade. Despite the fact that other major subtype B clades in Guatemala mostly circulate among individuals infected by the same transmission route (heterosexual transmission), the mean estimated growth rate of these lineages varied considerably, ranging from 0.2 year^-1^ for clade B_GU-III_ to 2.1 year^-1^ for clade B_CAM-I/GU-II_. This range is even higher than that estimated for country-specific B_PANDEMIC_ clades circulating in the general population in different Latin American countries (0.2–0.9 year^-1^) [7, 8, 36].

It is important to acknowledge some limitations of our study design. Even when the study sample size is large, sequences were obtained from a single HIV clinic. Additionally, the time frame for collecting HIV sequences was limited (2010–2013). Nevertheless, the Roosevelt Hospital is the largest of a total of 18 HIV clinics in the country, providing antiretroviral treatment for 30% of all registered HIV-infected persons in Guatemala [15], and the demographic characteristics (including female-to-male ratio, mean age, race, marital status, literacy) and geographical distribution of persons attending this clinic are highly representative of the whole population living with HIV in Guatemala [11, 16, 17]. Indeed, among the Roosevelt Hospital’s clients, 45% reside outside the department of Guatemala [14, 16]. Our study group is highly representative of the Roosevelt Hospital cohort, as more than 80% of all adults diagnosed at this clinic during the enrolment period were included in the study (expecting approximately 450 total new cases per year [16]). Given these characteristics of the Roosevelt Hospital’s cohort, we would not expect our main observations to vary significantly if additional participants enrolled in clinics from around the country were to be included.

## Conclusions

In summary, our observations suggest that the Guatemalan HIV-1 subtype B epidemic is being driven by dissemination of multiple B_PANDEMIC_ founder viral strains, some of them mostly restricted to Guatemala and others widely disseminated in the Central American region. Moreover, our results identify Guatemala and Honduras as hubs that have played a central role in dissemination of B_PANDEMIC_ viral strains within Central America and further suggest the existence of different sub-epidemics with distinct evolutionary and demographic dynamics within Guatemala and for which different targeted prevention efforts might be needed.

## Supporting information

S1 Data Selected HIV-1 subtype B PR-RT sequences. Fasta HIV-1 subtype B PR-RT Guatemalan sequences included in the study.
(TXT)

S1 Table Origin and sampling interval of HIV-1 subtype B sequences. (DOCX)

S2 Table Distribution of HIV-1 subtype B sequences of Central American countries in Guatemalan B_CAM_ clades. (DOCX)

S3 Table Best fit demographic model for the major HIV-1 Guatemalan clades. Log marginal likelihood (ML) estimates for the logistic (Log), exponential (Expo) and expansion (Expa) growth demographic models obtained using the path sampling (PS) and stepping-stone sampling (SS) methods. The Log Bayes factor (BF) is the difference of the Log ML between of alternative (H1) and null (H0) models (H1/H0). Log BFs > 3 indicates that model H1 is more strongly supported by the data than model H0.
(DOCX)
