# Supplementary material for: Evolutionary history and spatiotemporal dynamics of the HIV-1 subtype B epidemic in Guatemala
Source: PLoS One. 2018 Sep 13;13(9):e0203916. doi: 10.1371/journal.pone.0203916 (PMC6136800; doi:10.1371/journal.pone.0203916)
Supplement: S1 Table — (DOCX) [file pone.0203916.s002.docx]

**S1 Table. Origin and sampling interval of HIV-1 subtype B sequences.**

| **Region** | **Country** | **New sequences** | **Published sequences** | **Sampling interval** |
| --- | --- | --- | --- | --- |
| Central America | Guatemala | 1047 | - | 2010-2013 |
|  | Belize | - | 9 | 2004 |
|  | Costa Rica | - | 2 | 2002, 2007 |
|  | El Salvador | - | 170 | 2008-2010 |
|  | Honduras | - | 513 | 2001-2009 |
|  | Panama | - | 583 | 2004-2013 |
|  | Mexico | - | 824 | 2004-2010 |
| Caribbean | Dominican Republic | - | 78 | 2005-2010 |
|  | Jamaica | - | 70 | 2005-2010 |
|  | Trinidad and Tobago | - | 58 | 2000-2003 |
|  | Others | - | 32 | 2000-2005 |
| North America | United States | - | 465 | 1982-2010 |
| Europe | France | - | 344 | 1983-2008 |
