# Supplementary material for: Evolutionary history and spatiotemporal dynamics of the HIV-1 subtype B epidemic in Guatemala
Source: PLoS One. 2018 Sep 13;13(9):e0203916. doi: 10.1371/journal.pone.0203916 (PMC6136800; doi:10.1371/journal.pone.0203916)
Supplement: S2 Table — (DOCX) [file pone.0203916.s003.docx]

**S2 Table. Distribution of HIV-1 subtype B sequences of Central American countries in Guatemalan B_CAM_ clades^a^.**

|  |  | Guatemala | Belize | Costa Rica | El Salvador | Honduras | Panama^b^ | Mexico |
| --- | --- | --- | --- | --- | --- | --- | --- | --- |
|  |  | (n = 1047) | (n = 9) | (n = 2) | (n = 170) | (n = 513) | (n = 583) | (n = 824) |
| **B_CAM-I_** | (n = 599) | 197 (18.8) | 1 (11.1) | 0 | 14 (8.2) | 374 (72.9) | 0 | 13 (1.6) |
| **B_CAM-II_** | (n = 224) | 130 (12.4) | 1 (11.1) | 0 | 57 (33.5) | 34 (6.6) | 0 | 2 (0.2) |
| **B_CAM-III_** | (n = 150) | 86 (8.2) | 0 | 0 | 0 | 63 (12.3) | 0 | 1 (0.1) |
| **B_CAM-IV_** | (n = 126) | 104 (9.9) | 0 | 0 | 21 (12.4) | 0 | 0 | 1 (0.1) |
| Total |  | 517 (49.4) | 2 (22.2) | 0 | 92 (54.1) | 471 (91.8) | 0 | 18 (2.2) |

^a^Data are: number (%)

^b^Panamanian country-specific clades only were included in the analysis.
