# Supplementary material for: Evolutionary history and spatiotemporal dynamics of the HIV-1 subtype B epidemic in Guatemala
Source: PLoS One. 2018 Sep 13;13(9):e0203916. doi: 10.1371/journal.pone.0203916 (PMC6136800; doi:10.1371/journal.pone.0203916)
Supplement: S3 Table — Log marginal likelihood (ML) estimates for the logistic (Log), exponential (Expo) and expansion (Expa) growth demographic models obtained using the path sampling (PS) and stepping-stone sampling (SS) methods. The Log Bayes factor (BF) is the difference of the Log ML between of alternative (H1) and null (H0) models (H1/H0). Log BFs > 3 indicates that model H1 is more strongly supported by the data than model H0. (DOCX) [file pone.0203916.s004.docx]

**S3 Table.** Best fit demographic model for the major HIV-1 Guatemalan clades.

| **Clade** | **Model** | **PS** | **Models compared** | **Log BF** | **SS** | **Models compared** | **Log BF** |
| --- | --- | --- | --- | --- | --- | --- | --- |
|  |  | **Log ML** |  |  | **Log ML** |  |  |
| B_GU-I_ | **Expo** | **-4671.5** | **-** | **-** | **4671.5** | **-** | **-** |
|  | Log | -4672.7 | Expo/Log | 1.1 | -4673.1 | Expo/Log | 1.6 |
|  | Expa | -4675.4 | Expo/Expa | 3.9 | -4675.7 | Expo/Expa | 4.2 |
| B_GU-II_ | **Log** | **-2813.5** | **-** | **-** | **-2813.6** | **-** | - |
|  | Expo | -2816.6 | Log/Expo | 3.1 | -2816.6 | Log/Expo | 3.0 |
|  | Expa | -2817.9 | Log/Expa | 4.4 | -2818.0 | Log/Expa | 4.4 |
| B_GU-III_ | **Expo** | **-3348.9** | **-** | **-** | **-3349.1** | **-** | **-** |
|  | Log | -3350.1 | Expo/Log | 1.2 | -3350.5 | Expo/Log | 1.4 |
|  | Expa | -3352.7 | Expo/Expa | 3.8 | -3352.8 | Expo/Expa | 3.7 |
| B_GU-V_ | **Expo** | **-3926.5** | **-** | **-** | **-3926.7** | **-** | **-** |
|  | Log | -3927.8 | Expo/Log | 1.3 | -3928.0 | Expo/Log | 1.3 |
|  | Expa | -3928.2 | Expo/Expa | 1.7 | -3928.3 | Expo/Expa | 1.6 |
| B_GU-VI_ | **Log** | **-5127.0** | **-** | **-** | **-5127.1** | **-** | - |
|  | Expo | -5135.9 | Log/Expo | 8.9 | -5136.1 | Log/Expo | 9.0 |
|  | Expa | -5142.8 | Log/Expa | 15.8 | -5143.1 | Log/Expa | 16.0 |
| B_GU-VIII_ | **Expo** | **-4137.7** | **-** | **-** | **-4137.7** | **-** | **-** |
|  | Log | -4139.4 | Expo/Log | 1.7 | -4139.7 | Expo/Log | 2.0 |
|  | Expa | -4143.4 | Expo/Expa | 5.7 | -4143.6 | Expo/Expa | 5.9 |
| B_CAM-1/a_ | **Log** | **-4931.1** | **-** | **-** | **-4931.3** | **-** | **-** |
|  | Expo | -4936.5 | Log/Expo | 5.4 | -4936.5 | Log/Expo | 5.2 |
|  | Expa | -4946.8 | Log/Expa | 15.7 | -4947.2 | Log/Expa | 15.9 |
| B_CAM-I/b_ | **Log** | **-3942.7** | **-** | **-** | **-3943.0** | **-** | **-** |
|  | Expo | -3953.1 | Log/Expo | 10.4 | -3953.0 | Log/Expo | 10.0 |
|  | Expa | -3958.0 | Log/Expa | 15.3 | -3958.0 | Log/Expa | 15.0 |
| B_CAM-III_ | **Log** | **-5854.6** | **-** | **-** | **-5855.2** | **-** | - |
|  | Expo | -5856.4 | Log/Expo | 1.8 | -5856.6 | Log/Expo | 1.4 |
|  | Expa | -5863.9 | Log/Expa | 9.3 | -5864.4 | Log/Expa | 9.2 |
| B_CAM-IV_ | **Log** | **-10687.0** | **-** |  | **-10688.0** | **-** | **-** |
|  | Expo | -10693.9 | Log/Expo | 6.9 | -10694.1 | Log/Expo | 6.1 |
|  | Expa | -10703.6 | Log/Expa | 16.6 | -10703.7 | Log/Expa | 15.7 |

Log marginal likelihood (ML) estimates for the logistic (Log), exponential (Expo) and expansion (Expa) growth demographic models obtained using the path sampling (PS) and stepping-stone sampling (SS) methods. The Log Bayes factor (BF) is the difference of the Log ML between of alternative (H1) and null (H0) models (H1/H0). Log BFs > 3 indicates that model H1 is more strongly supported by the data than model H0.
